# Juvenile myoclonic epilepsy shows increased posterior theta, and reduced sensorimotor beta resting connectivity

**DOI:** 10.1016/j.eplepsyres.2020.106324

**Published:** 2020-07

**Authors:** Bethany Routley, Alexander Shaw, Suresh D Muthukumaraswamy, Krish D Singh, Khalid Hamandi

**Affiliations:** a Cardiff University Brain Research Imaging, School of Psychology, Cardiff University, United Kingdom; b School of Pharmacy, Faculty of Medical and Health Sciences, University of Auckland, Auckland, New Zealand; c The Wales Epilepsy Unit, Department of Neurology, University Hospital of Wales, Cardiff, United Kingdom

**Keywords:** JME, Connectivity, Juvenile myoclonic epilepsy, MEG, Magnetoencephalography

## Abstract

**Background:**

Widespread structural and functional brain network changes have been shown in Juvenile Myoclonic Epilepsy (JME) despite normal clinical neuroimaging. We sought to better define these changes using magnetoencephalography (MEG) and source space connectivity analysis for optimal neurophysiological and anatomical localisation.

**Methods:**

We consecutively recruited 26 patients with JME who underwent resting state MEG recording, along with 26 age-and-sex matched controls. Whole brain connectivity was determined through correlation of Automated Anatomical Labelling (AAL) atlas source space MEG timeseries in conventional frequency bands of interest delta (1−4 Hz), theta (4−8 Hz), alpha (8−13 Hz), beta (13−30 Hz) and gamma (40−60 Hz). We used a Linearly Constrained Minimum Variance (LCMV) beamformer to extract voxel wise time series of ‘virtual sensors’ for the desired frequency bands, followed by connectivity analysis using correlation between frequency- and node-specific power fluctuations, for the voxel maxima in each AAL atlas label, correcting for noise, potentially spurious connections and multiple comparisons.

**Results:**

We found increased connectivity in the theta band in posterior brain regions, surviving statistical correction for multiple comparisons (corrected p < 0.05), and decreased connectivity in the beta band in sensorimotor cortex, between right pre- and post- central gyrus (p < 0.05) in JME compared to controls.

**Conclusions:**

Altered resting-state MEG connectivity in JME comprised increased connectivity in posterior theta – the frequency band associated with long range connections affecting attention and arousal - and decreased beta-band sensorimotor connectivity. These findings likely relate to altered regulation of the sensorimotor network and seizure prone states in JME.

## Highlights

• We investigated whole brain source space connectivity in JME using across standard MEG frequency bands.
• Connectivity was increased in posterior theta and alpha bands in JME, and decreased in sensorimotor beta band.
• Our findings highlight altered interactions between posterior networks of arousal and attention and the motor system in JME.

## 1 Introduction

Juvenile Myoclonic Epilepsy (JME) is one of the most common epilepsy syndromes. The underlying basis for JME remains unknown. JME is a sub-syndrome of the Genetic Generalised Epilepsies (GGE) (previously known as Idiopathic Generalised Epilepsy), alongside Juvenile Absence Epilepsy (JAE), Childhood Absence Epilepsy (CAE), and Epilepsy with Generalised Tonic Clonic Seizures Only (EGTCS) (Wolf and Beniczky, 2014). JME is a lifelong condition usually presenting in the second decade of life with myoclonic jerks (MJ), absence seizures and generalised tonic-clonic seizures. Seizures are linked to states of arousal, typically occurring in the first hour after waking and are more likely after sleep deprivation (Panayiotopoulos et al., 1994). MRI brain scans are normal to visual inspection, and there are no other structural, biochemical or metabolic clinical markers in JME. Clinical electroencephalography (EEG) in JME is characterised by normal background rhythms, and abnormal interictal spike and polyspike wave discharges with frontal predominance (Camfield et al., 2013), and photosensitivity is present in around 30 % of people with JME (Panayiotopoulos et al., 1994).

Several recent lines of evidence show JME as a brain network disorder with predominantly frontal, but also parieto-occipital and sub-cortical involvement. Neuropsychological profiles show alterations in executive, frontal lobe function (Chowdhury et al., 2014a). Frontal lobe changes are also seen with quantitative analysis of structural MRI (Focke et al., 2014; Vollmar et al., 2012); and frontal ‘hyperconnectivity’ has been shown with fMRI and DTI analyses (Caeyenberghs et al., 2015; Vollmar et al., 2012; Vulliemoz et al., 2011; Wandschneider et al., 2012). In addition grey and white matter changes beyond the frontal lobes are also reported (Alhusaini et al., 2013; Caeyenberghs et al., 2015; Kim et al., 2015). Source localisation of spike wave discharges (SWD) implicates early involvement of mesial frontal structures in their genesis (Holmes et al., 2010; Stefan et al., 2009), and EEG-fMRI studies show involvement of ‘default mode’ and subcortical brain areas during SWD (Aghakhani et al., 2004; Gotman et al., 2005; Hamandi et al., 2006), with the precuneus ‘driving’ or modulating these discharges (Vaudano et al., 2009).

EEG and magnetoencephalography (MEG) are suited to study whole brain network activity on a time scale similar to cognitive and epileptic processes. Previous neurophysiological studies in JME have been mostly restricted to those using EEG. An early study comparing the spectral profiles in resting rhythms at EEG sensor derivations in the 3 sub-syndromes, JAE, JME, and EGTCS, compared to controls, found increases in delta, theta and alpha power and decreased beta power in all GGE groups; and increased frontal delta, and global reduction in beta power in JME (Clemens et al., 2000). A further study, using low-resolution brain electromagnetic tomography (LORETA) source localisation of scalp EEG in a similar population, found increased theta source density in the posterior cortex in JME, compared to controls, and decreased source density at 20 Hz in parietal areas, parietal lobule and posterior cingulate (Clemens et al., 2012). A graph-theoretic analysis between EEG epochs in an immediate pre-ictal state (immediately before generalised spike wave discharge (GSW)), and interictally, at least 10 s from the next GSW, found increased functional connectivity in delta, theta and an alpha1 (7.5–10 Hz) band, and decreases in alpha2 (10.5–12 Hz) and beta band functional connectivity compared to normal controls; these measures were all increased in the immediate pre-ictal state (Clemens et al., 2013). Others found increased connectivity, in a sensor-space analysis, in a 6–9 Hz frequency band in GGE, but not other bands tested between 1 and 70 Hz (Chowdhury et al., 2014b); and higher EEG source power in frontal theta and alpha bands (Tikka et al., 2013).

We are aware of only one MEG study that reported on network connectivity differences between patients with JME and heathy controls, and a focal epilepsy group. This study found significant increases in total power in all bands except the 8−12 Hz band in JME versus controls, and that JME ‘presented greater efficiency’ - a measure of how well the network exchanges information - and ‘lower eccentricity’ ie lower path distance, than the control subjects for the beta and gamma, without a clear topography’ (Niso et al., 2015).

Here, we sought to identify connectivity differences between patients with JME and controls using resting-state MEG, combining both the high temporal resolution of MEG, but also the spatial anatomical localisation of whole head MEG to a cortical anatomical parcellation atlas. We adopted the methods of (Colclough et al., 2015) using MEG connectivity measures based on spectral features to provide insights into the large-scale organisation of brain activity; this was done through the correlation of the temporal evolution of spectral power over a five minute resting MEG recording across classical frequency bands between different brain regions. We used the amplitude envelope correlation instead of phase based measures as this has been found to be the most consistent method for stationary connectivity estimation in MEG recordings; whereas phase- or coherence-based metrics such as the phase lag index or the imaginary part of coherency show poor test-retest reliability in (Colclough et al., 2016).

## 2 Methods

### 2.1 Patient selection

Patients with a diagnosis of JME were recruited prospectively from specialist epilepsy clinics at University Hospital of Wales, Cardiff. Inclusion criteria included seizure onset in late childhood or adolescence, with myoclonic jerks, with or without absence seizures, and generalised tonic-clonic seizures; normal childhood development as assessed on clinical history; generalised spike wave on EEG and normal structural MRI. Patients were selected from a larger body of work of structural and functional imaging in epilepsy, some of which is published elsewhere (Caeyenberghs et al., 2015; Hamandi et al., 2011). Data from 26 patients with JME aged 18–48 (median 27), 7 males, in whom an eyes open resting state MEG was performed were analysed along with 26 age and gender matched controls. Patient studies were approved by the South East Wales NHS ethics, and Cardiff and Vale Research and Development committees, and the 100 Brains project from School of Psychology, Cardiff University Ethics Committee, Cardiff, UK. All participants gave written informed consent.

### 2.2 MEG acquisition

Whole-head MEG recordings were made using a 275-channel CTF radial gradiometer system. An additional 29 reference channels were recorded for noise cancellation purposes and the primary sensors were analysed as synthetic third-order gradiometers (Vrba and Robinson, 2001). Two or three of the 275 channels were turned off due to excessive sensor noise (depending on time of acquisition). Subjects were seated upright in the magnetically shielded room. To achieve MRI/MEG co-registration, fiduciary markers were placed at fixed distances from three anatomical landmarks (left/right preauricular and nasion) prior to the MEG recording and identifiable on the subject’s anatomical MRI, and their locations were verified afterwards using high-resolution digital photographs. Head localisation was performed before and after each recording. Recordings were made at either 600 Hz or 1200 Hz. All recordings were later downsampled to 600 Hz for analysis and the data were analysed in synthetic third order gradiometer mode. For the 5-minute eyes-open rest recording, subjects were asked to sit comfortably in the MEG chair while their head was supported with a chin rest and with eyes open focus on a red dot on a grey background. Displays were generated in MATLAB® (The MathWorks, Inc.), using the Psychophysics Toolbox extensions (Brainard, 1997), and were presented on a Mitsubishi Diamond Pro 2070 monitor (1024 768 pixel resolution, 100 Hz refresh rate).

### 2.3 MRI acquisition

A GE HDx 3 T scanner (GE Healthcare, Milwaukee WI) was used for all MRI acquisition. An axial 3D fast spoiled gradient recalled (FSPGR) sequence was acquired (TR/TE/TI = 8/3/ 450 ms; Flip Angle = 20°; acquisition matrix = 256(AP)x192(LR)x172(SI), 1 mm isotropic voxels) for MEG co-registration.

### 2.4 MEG analysis overview

**Fig. 1:**
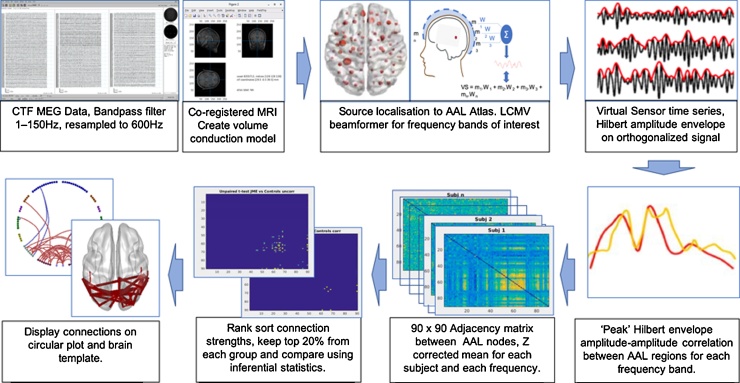
Visual schematic of the processing steps of the source space spectral and connectivity analysis and comparison between groups.

MEG generates multi-dimensional data, which can be analysed in a large variety of ways. We sought to conduct an analysis investigating only consistent functional connectivity across the brain, while reducing noise. We focused on amplitude-amplitude connectivity of beamformer-derived oscillatory source signals, one of the most robust and repeatable types of MEG connectivity measures (Colclough et al., 2015; Koelewijn et al., 2019). Connectivity was assessed across six frequency bands based on standard nomenclature and convention: delta 1–4, theta 4–8, alpha 8–13, beta 13–30, low gamma 40–60, and high gamma 60–80 Hz; and between 90 Automatic Anatomical Labelling (AAL) atlas (Tzourio-Mazoyer et al., 2002) brain regions (Koelewijn et al., 2019). Fig. 1 shows a visual schematic of the processing steps of the source space spectral analysis.

### 2.5 Data pre-processing

MEG data was band-pass filtered between 1 and 150 Hz. The 5-minute resting datasets where then segmented into 2 s epochs. All data was visually inspected and any 2 s epochs containing major motion, muscle or eye-blink artefact, or interictal spike wave discharges were excluded from subsequent analysis. Co-registration was performed manually between MEG and MRI, with MEG fiducial locations marked manually on the participants MRI for nasion, left ear and right ear.

### 2.6 Source space localisation

The MEG sensor data were source-localised using FieldTrip (RRID:SCR_004849) version 20,161,011 (Oostenveld et al., 2011) with an LCMV beamformer. Leadfields were calculated using a localspheres forward model using a brain surface derived from segmented MRI. The trial average covariance matrix was constructed for each of the 6 frequency bands of interest. For each band, the beamformer weights were normalized by the vector norm, data were normalized to the MNI template, and reduced to 90 nodes following the Automatic Anatomical Labelling (AAL) atlas (Tzourio-Mazoyer et al., 2002). Epochs were then concatenated to generate a continuous virtual-sensor timecourse for each voxel. Each of the 90 AAL parcels was represented by a single voxel, which was selected based on it having the largest temporal standard deviation of all voxels in that AAL region (Koelewijn et al., 2019).

The resulting 90-node time series of per AAL region virtual sensors were orthogonalized to avoid spurious correlations using a multivariate regression approach known as symmetric orthogonalisation (Colclough et al., 2015). Oscillatory amplitude envelopes for each of these 90 concatenated and orthogonalized virtual sensor time series were obtained by performing a Hilbert transform to yield the analytic signal. These envelopes were subsequently despiked to remove artefactual temporal transients using a median filter, downsampled to 1 Hz and trimmed to avoid edge effects (removing the first 2 and last three samples) (Koelewijn et al., 2019).

### 2.7 Source space connectivity analysis

An amplitude correlation matrix was calculated based on the Pearson correlation between each AAL node’s Hilbert envelope for each subject and frequency band. Correlation coefficients were adjusted for the non-linearity of Pearson's R by the common Fisher transform to allow averaging and statistical testing. The aim of the Fisher Z transformation is to normalise the distribution of the correlation coefficients, with variance that is stable across different values. This procedure per se is therefore to standardise the distribution for subsequent statistical testing and does not deal with unequal variance between the groups. These Z scores were then converted to an adjusted Z-statistic using the estimated temporal degrees of freedom in each dataset, to account for the fact that different subjects may have had connectivity assessed over concatenated timeseries of different lengths due to our artefact epoch rejection step. Finally, these Z-statistic images were corrected for global effects by Z-scoring so that each subject’s connectivity map had zero mean and unit standard-deviation. This final procedure corrects for any systematic per-subject differences in sensitivity due to methodological confounds (Siems et al., 2016; Yan et al., 2013).

These corrected connectivity matrices were used to statistically compare the between node connectivity at each frequency band between JME patients and controls. As is common in network analysis, weighted edge-connectivity maps were thresholded to suppress connections considered to be “noise” rather than “signal”. This was done by selecting AAL connections which were robustly present across the cohort by ranking the connections for each group in order of strength, here the strongest connection was given the value 1 and the weakest given value 0. ‘Valid connections’ were taken as those with an average rank above a threshold of 0.8, indicating that these connections are consistently among the strongest across participants (Koelewijn et al., 2019). Importantly, a connection was taken forward for our analysis if it was seen as “valid” in either a JME or Control participant.

In addition to analysing effects within each frequency band separately, we also calculated a combined measure of connectivity by calculating the vector-sum of all connectivity matrices (the combined correlation matrix is based on summing and squaring the Pearson correlation coefficients) from each frequency band (Koelewijn et al., 2019). The objective being to maximise the amount of information used to assess group differences; using the following formula for each element (i,j) in the square connectivity matrix:

$${Combined}_{i,j}=sqrt(\;{{Delta}_{i,j}}^{2}+\;{{Theta}_{i,j}}^{2}+\;{{Alpha}_{i,j}}^{2}+{{Beta}_{i,j}}^{2}+\;{{Low\;Gamma}_{i,j}}^{2}+{{High\;Gamma}_{i,j}}^{2})$$

We tested the difference between JME patients and controls using an unpaired *t*-test of the corrected Z-scores, at each of the selected “valid” (top 20 %) connectivity edges and looked for significant edges at both p < 0.05 (uncorrected) and at p < 0.05 (corrected using a 10,000-permutation test with omnibus thresholding).

### 2.8 Source space activity

In order to assess possible cohort differences in oscillatory activity, rather than connectivity, we also calculated a measure of temporal variance at each beamformer-reconstructed voxel in the brain, for each separate frequency band, via an assessment of the amplitude envelopes. Due to large variations in signal sensitivity throughout the brain, beamformer reconstructions of virtual-sensors result in signals in which the mean and temporal standard-deviation of the amplitude envelopes are highly correlated and show large variations across the source space. For that reason, we choose to use a normalized measure, the coefficient of variation, which is the simply the standard-deviation of the amplitude envelope divided by the mean of the amplitude envelope – i.e. a measurement of the degree to which the activity in a given region fluctuates around its mean amplitude over time. For each AAL region, statistical differences were assessed between JME and control cohorts separately for each frequency band using randomisation-based independent t-tests with 5000 permutations and omnibus correction for multiple comparisons (Nichols and Holmes, 2002). Effects are rendered on a template AAL90 brain parcellation.

### 2.9 Sensor space power

We also examined the power spectra of various frequency bands in sensor space to compare with the existing literature. Using the FieldTrip toolbox (Oostenveld et al., 2011) the pre-processed data were first converted to planar gradient formation and frequency analysis was conducted using Hanning-windowed fast Fourier transforms. The gradients over both planar directions were then combined to obtain a single positive-valued number under each sensor. In this sensor configuration, sources can be assumed to lie directly underneath local maxima on field maps, thus allowing the results of this analysis to be more easily interpretable (Bastiaansen and Knösche, 2000). Frequency analysis was conducted between 1 and 30 Hz in 0.5 Hz frequency steps to give local maximal under the sensors. We compared differences in sensor level power between JME and control cohorts in the following frequency bands, delta (1–4 Hz), theta (4–8 Hz), alpha (8–13 Hz), beta (13–30 Hz), using a 2-sample *t*-test. Statistical differences between the JME and control cohorts were determined using randomisation testing of these difference images (5000 permutations, omnibus correction for multiple comparisons).

## 3 Results

### 3.1 Participants

A summary of clinical characteristics of the 26 patients with JME are given in Table 1 (and full details in Supplementary Table, 1s). Patients and controls were matched for age and gender, patients (19 F/7 M median age 27 ± 8, handedness 25 right, 1 left), control subjects (19 F/7 M, median age 26.5 ± 7, handedness 26 right). Median duration since onset of first seizures in patients was 14 years, all patients were taking anti-epileptic drugs (AED).

**Table 1:** Summary of patient and control characteristics. (see table s1, supplementary material for individual patient characteristics). MJ – myoclonic jerks, GTCS – generalised tonic clonic seizures, LEV- levetiracetam, VPA- sodium valproate, LTG- lamotrigine, TPM-topiramate, ZNM-zonisamide. PPR – photoparxysmal response. (see table s1 for individual patient characteristics).

	JME		Controls
**Age**	27 ± 8		26.5 ± 7
**Sex**	19 F/7 M		19 F/7 M
		**Age onset, median (range)**	
**Seizures (number of patients),**	MJ (N = 26, 100 %)	15 (8−20)	
	Absences (N = 15)	14 (8–18)	
	GTCS (N = 26, 100 %)	15 (7–24)	
**PPR on EEG**	N = 8 (30 %)		
**Number of AEDs**	12 monotherapy 10 on 2 drugs 4 on 3 drugs		
**AED name and number taking each**	LEV, N = 13 VPA, N = 12 LTG, N = 5 TPM, N = 4 ZNM, N = 4		

### 3.2 MEG

All patients and controls had good quality resting-state MEG recordings suitable for further analysis. Following artefact rejection, there were no significant group differences in the number of remaining trials (controls mean 146 ± 4.5, patients mean 142 ± 12.1; t(50) = 1.7, p = 0.09). Only two patients had generalised spike wave during the 5-minute resting recording, one with 4 episodes under 1 s, and the other with 2 episodes under one second; these epochs were marked and excluded from the subsequent analysis pipeline.

### 3.3 Whole brain functional connectivity

**Fig. 2:**
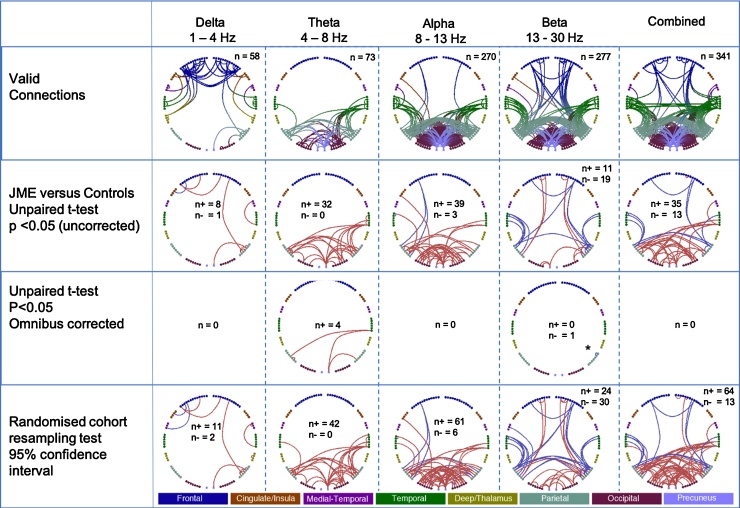
Comparison between JME group (N = 26) and Control group (N = 26) for each frequency band of interest and combined across all frequency bands, shown in each column, with results by row. showing 1) valid connections, those present after rank sort with threshold >0.8 for each group.. Anatomical regions and connections are colour coded, shown along the bottom bar. For subsequent rows connections that are decreased in JME are shown in blue, while connections that are increased in JME are shown in red. 2) Unpaired *t*-test (uncorrected), 3) Unpaired *t*-test, with omnibus correction - *the significant connection is between right pre- and post-central gyri, the pre-central AAL node placed with parietal nodes to better represent the anatomical distribution, and 4) after randomised cohort resampling.

**Fig. 3:**
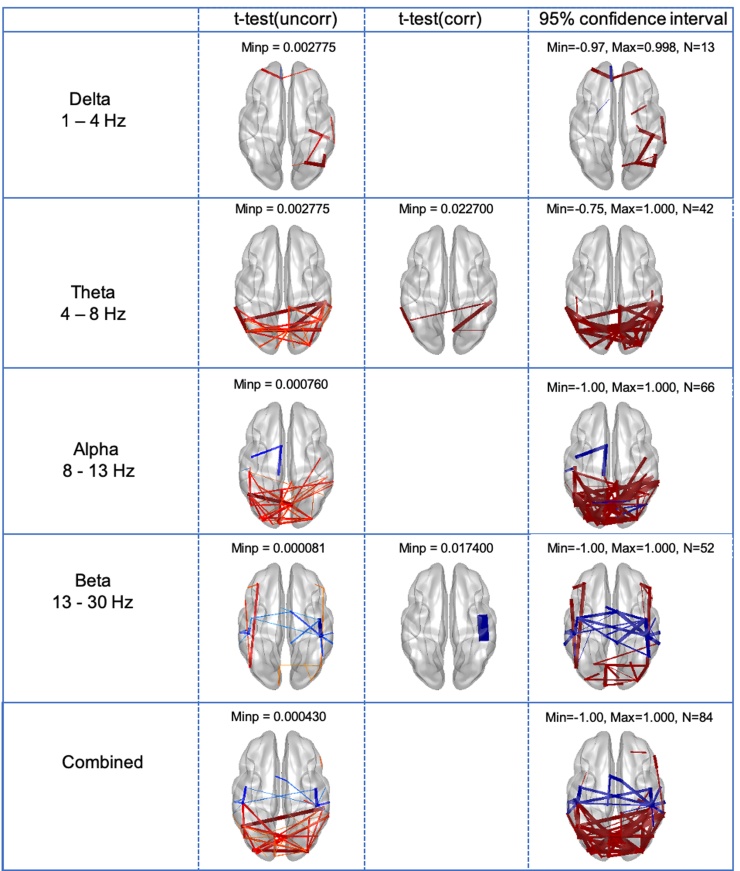
The same results as Fig. 2 shown on a template brain to better visualize the anatomical relationship of differences between JME and control groups. Comparison between JME and Controls for each frequency band of interest and combined across all frequency bands in each row, and columns showing uncorrected *t*-test, corrected *t*-test and 95 % confidence intervals.

**Fig. 4:**
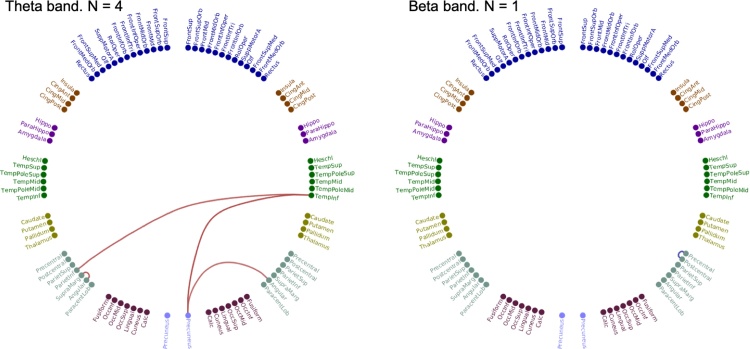
Circle plot of AAL nodes, with anatomical labels, and significant differences between JME and controls, unpaired *t*-test with omnibus correction, p < 0.05. The significant connection in the right panel is between right pre- and post-central gyri; the pre-central AAL node placed with parietal nodes to better represent the anatomical distribution.

We tested for differences in whole brain connectivity between AAL atlas regions based on an amplitude correlation of source time-series data from MEG, between patients with JME and controls, across classical EEG/MEG frequency bands. Fig. 2, Fig. 3, Fig. 4 to show the findings of the connectivity analysis for each of the frequency bands of interest and that of all frequency bands combined through each of the statistical tests applied.

We start by showing the number of valid connections for each frequency (the top 20 % of connections after a rank sort of all connections based on connection strength at the group level, in each group (JME and controls separately) as described in the methods). This rank sorting and selection criteria resulted in predominantly frontal connections in the delta band, posterior connections in the theta and alpha bands and both frontal and posterior connection in the beta band. No valid connections were found in the low gamma band and only two connections in the high gamma band (not shown here), and these frequency bands were not considered further. A two-sample *t*-test found differences between JME and controls across all four frequency bands, delta, theta, alpha and beta, predominantly with increased connectivity in JME in posterior brain regions for the theta and alpha band, and a combination of increased and decreased in between frontal, temporal, parietal and occipital in the beta band. The increased posterior theta connectivity in JME compared to controls, and decreased beta connectivity between right sensorimotor nodes were statistically significant after correction using the omnibus-corrected threshold, running 10,000 sign-shuffling iterations, and recomputing the t-statistic and p-values. In the theta band increased connectivity was found between the following AAL nodes – right inferior temporal gyrus and right precuneous, right inferior temporal gyrus and right inferior parietal gyrus, right angular gyrus and right precuneous; and left supramarginal gyrus and left angular gurus, and reduced connectivity in the beta band between right pre-central and right post central gyri (Fig. 4 and Table 2).

**Table 2:** AAL regions showing statistically significant differences between patients with JME and Controls for the theta (4 – 8 Hz) and beta (13 – 30 Hz) frequency bands. Regions and t-statistic, and p-values are shown, 2 sample *t*-test, JME vs controls, for the frequency bands and regions reaching statistical significance following omnibus correction for multiple comparisons (see method and Figs. 2–4).

Frequency band	AAL connections		t-statistic	p value
Theta, 4–8 Hz	R Inferior Temporal Gyrus	L Inferior Parietal Gyrus	3.7469	0.0344
	R Inferior Temporal Gyrus	R Precuneus	3.9000	0.0426
	R Angular Gyrus	R Precuneus	3.6631	0.0266
	L Angular Gyrus	L Supramarginal Gyrus	3.8272	0.0266
Beta, 13–30 Hz	R Postcentral Gyrus	R Precentral Gyrus	−4.3508	0.0155

We tested a "confidence interval" on the generalisability of the results. Across 10,000 iterations, half of one group is subsampled randomly and compared to a random half of the second group. Increases and decreases in connectivity are tabulated, and connections tested showing a consistent effect direction across 95 % of iterations are considered "robust" and plotted (blue for decreases, red for increases). This test does not take into account the magnitude of the effect, only its direction and how consistent this is across re-samplings. The findings from this randomised cohort resampling show predominant increased connectivity in posterior connectivity in JME in both the theta and alpha bands and in the beta band a combination of decreased connectivity between fronto-temporal-parietal nodes, and increased connectivity between frontal-parietal-occipital nodes.

### 3.4 Analysis of activity differences

**Fig. 5:**
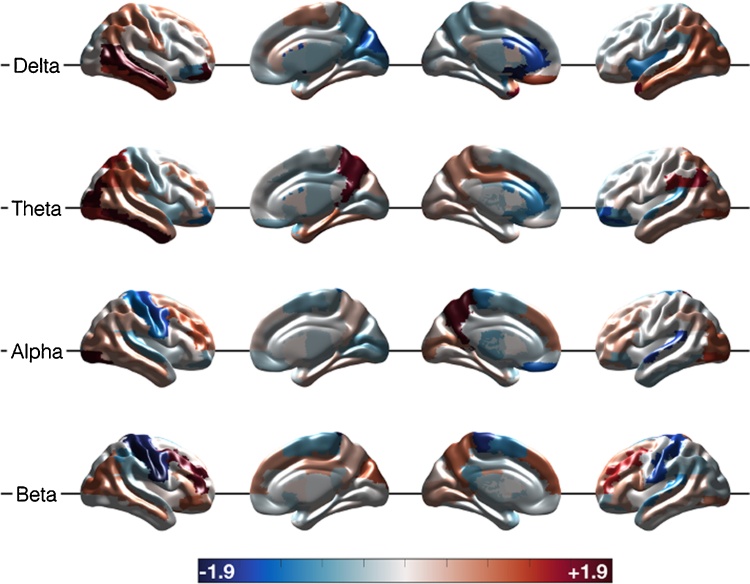
Comparison of ‘source activity’ between JME group (N = 26) and Control group (N = 26) for each frequency band of interest, unthresholded. Colour bar shows the mean change from the control mean, increases in JME compared to controls in red and decreases in JME compared to controls in blue. Changes jn right mid temporal delta only survived correction for multiple comparisons.

We compared differences in band limited activity (the standard-deviation of the amplitude envelope divided by the mean of the amplitude envelope as described in the methods) between patients and controls. There were widespread differences with this activity measure with increases in temporal and parietal delta activity, posterior theta activity, and decreases in sensorimotor beta activity, but also increases in frontal beta activity (Fig. 5). The only significant change after permutation testing was in the delta band in the R Mid Temp AAL atlas region t = 3.7, p = 0.039.

### 3.5 Analysis of sensor level in power

Significant differences were seen in a small number of right temporal sensors in the delta band, theta and alpha bands and in right frontal sensors in the beta band, with increased power in these sensors in JME compared to controls (supplementary Fig. 1).

## 4 Discussion

We investigated the differences in brain connectivity using MEG recorded resting brain activity between patients with JME and age- and gender- matched control subjects. We found statistically significant increases in connectivity in theta and alpha bands in posterior brain regions, and decreased connectivity in pre- and post- central (sensorimotor) brain regions. Our findings build on and corroborate previous reports using scalp EEG (Clemens et al., 2013) with a greater anatomical specificity given by using MEG source space analysis.

We also tested the 95 % confidence limit across all connections (uncorrected) to test the generalisability of our results in terms of the direction of an increase or decrease in connection strength between the two groups. We show the 95 % confidence interval test alongside the corrected and uncorrected *t*-tests, as it reveals a set of connections that are robustly increased, or decreased, in JME compared to controls, irrespective of the specific individuals entered into the test. The corrected and uncorrected *t*-test give a clearer depiction of potentially the most important differences between JME and control connections.

The classical neurophysiological frequency bands (Kane et al., 2017) tested here represent different local and global state changes in the brain. The delta band predominates during slow wave sleep or pathological states during wakefulness, but also increased in frontal areas during working memory tasks in healthy volunteers (Harmony, 2013). The theta and alpha bands are functions of arousal, attention and working memory - an area impaired in patients with JME (Pascalicchio et al., 2007) - and represent long range interactions (Palva and Palva, 2011). The higher frequencies (beta and gamma) predominate during local stimulus or saliency processing in more focal network activity (von Stein and Sarnthein, 2000v). The work of Clemens et al. and others shows altered theta power and connectivity as potential markers of JME (Clemens et al., 2000, Clemens et al., 2007, Clemens et al., 2011, Clemens et al., 2013; Elshahabi et al., 2015). Altered resting posterior EEG oscillations in the 6–9 Hz theta band have also been proposed as a potential disease classifier by others (Schmidt et al., 2016); using a short, 20 s segment of routine clinical EEG they found that a functional network inferred from the EEG of each individual subject, integrated with an oscillator, best distinguished patients with GGE from controls (Schmidt et al., 2016).

Posterior brain regions have also been implicated in the pathophysiology of JME from EEG-fMRI studies of generalised spike wave discharges, where GSW associated fMRI deactivation are seen in parietal lobes and precuneous, as well as mesial frontal regions, as part of the ‘default mode’ brain network (Gotman et al., 2005; Hamandi et al., 2006). Analysis of causal influence between these areas on spike wave discharge in two independent studies found the signal from the precuneous to be the driver or influence of the other regions (Lee et al., 2014; Vaudano et al., 2009). Our results here show increased node connectivity involving right precuneous in 2 of the 4 theta band connections surviving multiple comparison between JME and controls. The left and right angular gyrus both showed increased theta connectivity to precuneous and supramarginal gyrus. The angular gyrus forms part of the parietal association cortex, and is a major connecting hub shown in previous functional imaging connectivity studies that has a role in attention, memory, visual and semantic processing (Seghier, 2013). Whilst existing literature attributes psychological profiles in JME as a disorder of predominantly frontal lobe dysfunction (Motamedi et al., 2014; Piazzini et al., 2008; Wandschneider et al., 2012), our findings here, along with others, provide strong evidence for dysfunction outside the frontal lobe and in particular in posterior attentional networks and temporo-parietal regions (Caeyenberghs et al., 2015; Kim et al., 2015; Pascalicchio et al., 2007; Tian et al., 2010).

Beta band oscillations predominate in pre- and postcentral gyri along with alpha/mu rhythms (Groppe et al., 2013) with beta rhythms proposed to have a key role in sensorimotor regulation (Engel and Fries, 2010; Ritter et al., 2009) and orchestrate sensorimotor activity, motor planning and execution (Engel and Fries, 2010; Pfurtscheller, 1981). We have previously shown alterations in beta band task related responses in JME (Hamandi et al., 2011) and in benign epilepsy with centrotemporal spikes (BECTS) (Brindley et al., 2016; Koelewijn et al., 2015). Altered connectivity and oscillatory activity in both these conditions likely reflect pathological alterations leading to the clinical manifestations of myoclonic jerks in JME and focal motor seizures in BECTS. These measures may reflect an uncoupling of sensorimotor integration in JME. Others, using Transcranial Magnetic Stimulation, have shown reduced sensorimotor intracortical inhibition in JME (Manganotti et al., 2004)

Brain network dysfunction in epilepsy is also seen using fMRI and simultaneous EEG-fMRI studies (Centeno and Carmichael, 2014). One study found no changes in fMRI connectivity in areas seeded from GSW associated activation (Moeller et al., 2011), though others more recently have shown altered fMRI connectivity in JME affecting widespread cortical and subcortical areas - thalamus, cerebellum, precuneus, inferior temporal lobe and sensorimotor-related areas, including the middle cingulate cortex, supplemental motor area, and paracentral lobule (Qin et al., 2019; Zhong et al., 2018). In a task based fMRI study, increased functional connectivity between the motor system and frontal and parietal lobes in a working memory paradigm are seen with increased cognitive load (Vollmar et al., 2011), and also seen in first degree relatives of patients with JME (Wandschneider et al., 2014). These studies also showed less task-based fMRI deactivations in precuneus and medial prefrontal areas in JME relatives compared to controls, supporting a key link between cognitive and motor networks in JME. We did not find significant differences between patients and control in sub-cortical connectivity despite current evidence and hypotheses of thalamic and basal ganglia involvement in JME. This is likely due to the lower signal to noise of deeper sources recorded by MEG.

Our findings of decreased connectivity in sensorimotor cortex is in contradiction to that of Elshahabi et al. (2015), where they showed increased functional connectivity in alpha and beta bands in motor areas. Their study however differed from ours in terms of the diagnoses and age range of the cohort studied and the connectivity measures used. In their study of 13 patients included with IGE/GGE only one had JME, the others having other IGE subsyndromes JAE, CAE and GTCS only. There may be key differences in these neurophysiological makers between sub-syndromes and where possible they should be considered separately in future studies to better appreciate similarities or differences between them. Furthermore, the connectivity measure used the imaginary coherence between network nodes to generate connectivity matrices that were used to generate graph theory metrics of network connectivity. The study by Caeyenberghs et al. (2014) assessed structural rather than functional connectivity using DTI measure and a graph theory analysis, the relationship between structural and functional connectivity remain to be clarified, and negative correlations have been seen between structural and functional measures (Cociu et al., 2018).

We also looked for differences in a measure of source level activity (fluctuation in the Hilbert envelope of the virtual sensor timeseries) and sensor level power in each of the 4 frequency bands used in the connectivity analysis. The activity measure (calculated as standard deviation / mean) is a measurement of the degree to which a given region fluctuates around its mean amplitude over time. It is possible for a region to differ in its activity pattern to its connectivity pattern. This distinction is important when contrasting between groups. For example, it is possible to see increased connectivity between regions A and B in the JME group vs. controls, even though the activity of regions A or B may be reduced in the JME group compared to controls, and vice versa. Thus, activity (not power) is a complementary measure to functional connectivity. In contrast to activity, sensor power (the amplitude squared) gives a measure of the total signal strength within a specified frequency window for a sensor. Methodological issues with beamforming make it difficult to estimate power in source space (Luckhoo et al., 2014). We provide a comparison of band-limited power in sensor space and the activity measure in source space. The differences we found in source level activity and sensor level power are similar to those reported in the previous literature with overall increases in delta, theta and alpha frequency bands and decreased frontal beta band in JME compared to controls (Clemens et al., 2000).

There are several potential limitations to our study. Interictal epileptiform activity during the resting state recording could alter network connectivity dynamics in patients with JME (Qin et al., 2019). We manually inspected the data for movement and physiological artefacts, as well as epileptiform activity. We found a very low number of short lived GSW interictal discharges in only two patients. We excluded these GSW epochs from our analysis. This would exclude any artifactual differences in resting state rhythms between patients and controls in our study due to interictal paroxysms. We suspect several reasons for the low number of GSW events seen here. All our patients were taking AEDs, we acquired a 5-minute scan run where participants were seated in the MEG scanner, and asked to remain awake and fixate on a central cross-hair rather than relax fully; all scans were acquired during working hours after patients travelled to the centre, and the scanner environment was new to all our patients. GSW events are known to occur during relaxed wakefulness and more commonly in the first hour after waking, neither of which were met in our acquisition (Seneviratne et al., 2012).

Although we used a fairly homogenous group of patients with JME in terms of epilepsy presentation and classification, there was variation among the cohort in terms of seizure frequency, level of seizure control, duration of epilepsy and number and type of AEDs. Our patients were recruited from a tertiary epilepsy clinic and therefore reflect the more severe end of the spectrum in terms of seizure control. Most patients were taking more than one AED, the commonest AEDs here being levetiracetam, sodium valproate and lamotrigine. We were not able to control for AED use. Lamotrigine has been shown to decrease delta and theta power, and the level of lamotrigine-related power decrease correlated with initial (untreated) power (Zhong et al., 2018). Further, when compared with unmedicated patients, those treated with sodium valproate have shown a more 'normalised' functional connectivity, ie closer to controls (Clemens, 2008) compared to those not treated with sodium valproate. It may be the case that differences in power or connectivity were masked and/or exacerbated by a number of uncontrolled variables in our patient cohort, however the consistency of our findings with previous network alterations in JME, suggest a genuine disease specific effect here, rather than that related to treatments.

We have a relatively small sample size that precludes the analysis of correlations between connectivity measures and clinical parameters such as seizure frequency, severity and anti-epileptic drug type and doses, given the multiple clinical variables that can be generated. Nevertheless, the findings presented can form the basis for larger prospective studies where accurately ascertained and informed clinical variables can be compared against these connectivity measures.

A further limitation of our study is the use of the AAL atlas on which to base our virtual sensor locations for the connectivity analysis. A number of different atlases exist for the type of analysis conducted here. The purpose of the brain atlas being to reduce the data into meaningful parcellations that are computationally efficient and reflect underlying anatomy and physiology, for examples see (Fan et al., 2016; Wang et al., 2016). A detailed discussion and testing of different atlas selection is however beyond the scope of this paper. The AAL atlas is widely used in studies of functional MEG connectivity (Demuru et al., 2017; Jin et al., 2013; Koelewijn et al., 2019; Lardone et al., 2018; Velmurugan et al., 2019). In this context, the AAL regions are not assumed to work as single functioning units, but rather are based on a pragmatic choice, where regions of interest reflect cortical functional organisation across the brain, with adequate spatial separation to avoid signal leakage or cancellation with our beamformer methods, and that allows comparisons of brain structural and functional brain measures between individual and groups in a systematic way (Evans et al., 2012). A final methodological consideration is the thresholding of the connections, with only the 20 % strongest connections from each group taken forward for further analysis. This step is done to avoid analysing noise. We did not systematically test the choice of this threshold, which is outside the scope of this paper, nevertheless we chose a conservative estimate that has been used in other studies (Koelewijn et al., 2019), with resultant biologically plausible findings. A lower threshold would invariably lead to greater connections, some of which may be spurious, and a higher threshold likely too conservative.

### 4.1 Conclusion

Increases as well as decreases in connectivity in patients with epilepsy is in keeping with current concepts of ictogenesis, the process by which epileptic seizures develop. Increased posterior connectivity in theta and alpha (4−8 and 8−13 Hz) bands and decreased connectivity in sensorimotor beta (13−30 Hz) band may be the resting neurophysiological hallmark of JME and offer a potential biomarker for future studies of treatment effects and seizure risk.

## Declaration of Competing Interest

The authors declare no conflicts of interest.

We confirm that we have read the Journal’s position on issues involved in ethical publication and affirm that this report is consistent with those guidelines.
